# Relationship of regional myocardial deformation and myocardial fibrosis to myocardial trabeculation: The Multi-Ethnic Study of Atherosclerosis

**DOI:** 10.1186/1532-429X-18-S1-O77

**Published:** 2016-01-27

**Authors:** Nadine Kawel-Boehm, Robyn McClelland, Filip Zemrak, Gaby Captur, Gregory Hundley, Chia-Ying Liu, James Moon, Steffen E Petersen, Bharath Ambale Venkatesh, Joao A Lima, David A Bluemke

**Affiliations:** 1 grid.94365.3d0000000122975165National Institutes of Health, Bethesda, MD USA; 2 grid.452286.f0000000405113514Kantonsspital Graubuenden, Chur, Switzerland; 3 grid.34477.330000000122986657University of Washington, Seattle, WA USA; 4 grid.4868.20000000121711133Queen Mary University of London, London, UK; 5 grid.439632.9The Heart Hospital, London, UK; 6 Wake Forest, Winston-Salem, NC USA; 7 grid.21107.350000000121719311Johns Hopkins University, Baltimore, MD USA

## Background

A high degree of noncompacted (trabeculated) myocardium in relationship to compact myocardium (NC/C ratio >2.3) as measured by cardiac MR (CMR) has been associated with a diagnosis of left ventricular noncompaction (LVNC). However, a large proportion of healthy individuals fulfill the criterion of a LVNC; thus, the clinical significance of hypertrabeculated myocardium remains unknown. Purpose of this study was to determine if regional myocardial function and diffuse myocardial fibrosis are associated with the degree of LV trabeculation in a general population based cohort.

## Methods

The NC/C ratio was measured on steady-state free precession cine images in subjects from the Multi-Ethnic Study of Atherosclerosis "MESA5" follow-up cohort, free of clinical cardiovascular disease at baseline. Participants with myocardial scar on late-gadolinium enhancement imaging were excluded. Myocardial strain was measured as peak regional systolic circumferential shortening (Ecc) derived from CMR tagging images. LV myocardial T1 relaxation time, native and 12 and 25 min post contrast were determined by the T1 mapping technique and served as surrogate markers of myocardial fibrosis.

The association of LV trabeculation with strain (n = 1425 subjects) and T1 time (n = 1038 subjects) was assessed by linear regression in univariate and multivariate models adjusted for demographic variables, traditional cardiovascular risk factors and CMR measures of LV volume and function.

## Results

Mean, minimum and maximum NC/C ratio of all 9921 segments measured were 0.95, 0 and 5.4, respectively.

There was no association of the average NC/C ratio with global (average) Ecc in a per subject analysis in univariate and multivariate models (p > 0.05). In regional analysis, there was no association of the NC/C ratio with Ecc for the midventricular LV (p > 0.05) but a higher NC/C ratio was associated with a lower Ecc (i.e. better function) for the apical LV region in univariate analysis (B = -0.34%, p = 0.038). However, the association did not persist in adjusted models (p > 0.05).

In univariate analysis a higher average NC/C ratio was associated with a higher native T1 time (B = 2.9 ms, p = 0.015) but this did not persist in the adjusted models (p > 0.05). Uni- and multivariate analysis did not show an association between degree of trabeculation and T1 time measured at 12 or 25 min after contrast administration (p > 0.05).

## Conclusions

In a large population based cohort in individuals with a wide range of trabeculated myocardium, there was no consistent relationship between noncompacted myocardium as measured by the NC/C ratio and regional myocardial function or diffuse myocardial fibrosis. The absence of regional myocardial dysfunction or fibrosis may thus have the potential to discriminate patients with noncompaction from normal individuals with normal variant hypertrabeculation.

